# Knowledge, perceptions, and support for human milk banking among Saudi mothers: a cross-sectional study

**DOI:** 10.3389/fpubh.2026.1914184

**Published:** 2026-09-03

**Authors:** Yahya A. Almutawif, Renad M. Alhamawi

**Affiliations:** 1 Department of Clinical Laboratory Sciences, College of Applied Medical Sciences, Taibah University, Madinah, Saudi Arabia; 2 Health and Life Research Center, Taibah University, Madinah, Saudi Arabia

**Keywords:** cross-sectional study, donor human milk, human milk banking, maternal attitudes, preterm infants, Saudi Arabia

## Abstract

**Background:**

Donor Human Milk (DHM) is recommended for preterm infants when a mother’s own milk is unavailable. This study explored Saudi mothers’ knowledge, attitudes, and concerns regarding human milk banking (HMB) and donation, to identify barriers to acceptance and to inform future implementation.

**Methods:**

A cross-sectional survey of 835 mothers across Saudi regions assessed support for HMB and its association with sociodemographic factors, feeding practices, breastfeeding duration, and knowledge of the benefits of breast milk. Attitudes toward religious permissibility and willingness to accept or donate DHM were also evaluated.

**Results:**

Among 835 surveyed mothers, 37.7% supported HMB while 62.3% did not. Support was significantly higher among mothers aged 21–30 years (45.6% vs. 28.4% aged >40; *p* = 0.002) and those with one child (47.4% vs. 27.0% with >4 children; *p* < 0.001). Education and region showed no significant association with support (*p* > 0.05). Religious milk kinship concerns were the primary barrier, cited by 91.2% of non-supporters of HMB (*p* < 0.001); however, among participants who considered HMB permissible if the donor was known, 50.0% supported HMB. Furthermore, among participants who indicated that their stance could change after learning more about HMB and the benefits of human milk for preterm infants, 83.6% supported HMB, while 71.5% of those who would consider donating or receiving donor milk if breastfeeding were documented on a government platform supported HMB (*p* < 0.001). Logistic regression revealed that religious concerns about milk kinship (OR = 0.014; *p* < 0.001) and uncertainty about HMB legality (OR = 0.186; *p* = 0.033) were significant predictors of non-support.

**Conclusion:**

Overall, a considerable proportion of Saudi mothers demonstrated support for human milk banking (HMB) and willingness to accept or donate breast milk. However, religious concerns regarding milk kinship remain the primary barrier to wider acceptance. Successful HMB implementation requires three key strategies: (1) collaboration with religious scholars to establish secure donor–recipient registries addressing milk kinship concerns; (2) public education on safety and screening protocols; and (3) integration into national neonatal guidelines for preterm infants. The positive response to education and government-documented breastfeeding platforms suggests that culturally responsive, religiously informed approaches can significantly enhance HMB adoption and improve neonatal outcomes in Saudi Arabia.

## Background

Human milk (HM) is the optimal nutrition for infants’ development and protection, regardless of the gestational age (1). However, when maternal milk is unavailable, donor human milk (DHM) via wet nursing or human milk banks (HMB) is considered the best alternative, especially for preterm infants (1–3). In recent years, many countries have established HMB to provide HM for vulnerable neonates, particularly preterm and low-birth-weight infants (4, 5). This contributed to improving outcomes by reducing susceptibility to infection and providing nutrition that supports the immature immune systems of preterm infants (6).

In Saudi Arabia, approximately 25,000 infants are born prematurely (prior to 37 weeks) each year, with regional variation ranging from 7.5 to 8.4% in the central and eastern regions. (7–9). Preterm infants have different nutritional requirements for growth and immune development. Their vulnerable bodies and underdeveloped immune systems put them at risk of growth failure, developmental delays, and late-onset sepsis (10). Although breastfeeding is the cultural norm in Saudi Arabia, mothers of preterm infants face acute physiological and clinical barriers to milk expression during the early postpartum period, including delayed lactogenesis, emergency surgical deliveries, severe maternal morbidities (such as preeclampsia), and physical maternal–infant separation in the NICU. When the mother’s own milk is unavailable or insufficient, formula milk is the common alternative to feed preterm infants in hospital settings (4, 11, 12). While formula milk is the most common substitute, wet nursing remains a religiously and culturally accepted alternative in Saudi Arabia and is recognized within the Saudi Ministry of Health’s Policy of Breast Milk Substitutes in Hospitals (13). However, its actual prevalence in current clinical practice has not been quantified in published literature. Crucially, evidence consistently demonstrates that preterm infants fed HM, whether mother’s own or donor milk, exhibit a significantly lower incidence of necrotizing enterocolitis (NEC) compared to those fed milk formula (14). Furthermore, the establishment of HMB in neighboring and Muslim-majority nations, such as Iran, has demonstrated significant improvements in health outcomes among preterm infants, including reduced morbidity and mortality (15, 16). These findings highlight the essential role of human milk banks in improving neonatal survival and strongly support their implementation as a core component of hospital neonatal care. Therefore, providing such babies with HM is critical for the development and protection of preterm babies. Consequently, it is important to establish HMB in Saudi Arabia.

Despite increasing evidence supporting the benefits of human milk donation and the establishment of HMB, ethical dilemmas, cultural beliefs, lack of awareness, attitudes, and family concerns regarding the safety of donated milk continue to generate controversy (17–20). Religious considerations emerge as the primary hindrance to the establishment of milk banks and their shortcomings in Islamic nations (20–22).

In Saudi families, the practice of breastfeeding infants who are not biologically their own is deeply rooted, as they foster a sense of kinship by exchanging breast milk (23, 24). However, the idea of providing HM from an unfamiliar mother is not embraced within the community due to concerns about forming familial bonds that could potentially result in undesirable consequences, such as unintended sibling marriages (23, 25). This particular concern stands as the primary obstacle preventing the proposal and establishment of HMB in Saudi Arabia.

Evaluating the knowledge and attitudes of Saudi mothers who are predominantly Muslim is believed to be valuable in identifying the challenges they encounter when considering accepting donated milk for their infants in situations where breastfeeding is not possible. The main aim of this study was to explore the perspectives, beliefs, and concerns of Saudi mothers regarding HMB and to identify the challenges encountered by Saudi mothers in accepting donated or accepting milk for their infants. Also, another aim of this study was to assess breastfeeding practices and identify the factors associated with breastfeeding among mothers in Saudi Arabia. Understanding these practices and their determinants provides essential baseline evidence to support the planning and implementation of a human milk bank in hospitals. Specifically, these findings can help inform policies, guide donor recruitment strategies, identify potential barriers and facilitators to human milk donation and support the development of educational and public health initiatives that promote both breastfeeding and human milk donation. The outcomes of this research would serve as an initial foundation for informing policies, guiding donor recruitment strategies, identifying potential barriers and facilitators to human milk donation and the potential establishment of HMB in Saudi Arabia.

## Methods

### Study design

A retrospective cross-sectional study was conducted among Saudi mothers between April and July 2024. This design was selected to estimate the population prevalence of maternal awareness regarding HMB. During this period, an online questionnaire was distributed via WhatsApp (a widely used messaging platform among the Saudi population) and administered via Google Forms using a snowball sampling approach to reach mothers across multiple regions of Saudi Arabia.

### Participants and sample size determination

The study population comprised Saudi mothers aged 21 years or older with at least one child. Inclusion criteria were as follows: Saudi nationality, age 21 years or older, mother of at least one child regardless of the child’s current age, ability to read and comprehend Arabic, access to WhatsApp and the internet for online questionnaire completion and possession of a valid email address. Exclusion criteria included mothers unable to provide informed consent, those with cognitive or language barriers preventing questionnaire completion, and non-Saudis.

The sample size was calculated using the standard formula for estimating a population proportion: *n* = *Z*^2^*p*(1 − *p*)/*E*^2^. We used *Z* = 1.96 (for a 95% confidence level), *p* = 0.50 (a conservative estimate given the absence of prior prevalence data on maternal attitudes toward human milk banking in Saudi Arabia), and *E* = 0.035 (a 3.5% margin of error). This calculation yielded a minimum required sample size of *n* = 784 mothers.

### Data collection

The questionnaire was adapted from previously validated survey instruments that evaluate donor milk perceptions in similar socio-cultural contexts (26–29) and was modified to reflect Saudi socio-cultural norms and Islamic bioethical principles regarding milk kinship. A total of 21 closed-ended questions were organized into three sections: socio-demographic characteristics (5 items), lactation practices (4 items), and knowledge and attitudes toward milk donation, receiving donor milk, and human milk banks (12 items).

To ensure linguistic and cultural relevance, the questionnaire was developed and administered in Arabic. For reporting purposes, the final instrument was translated into English using a forward-and-back-translation process. In order to ensure content validity, scientific accuracy, and cultural relevance, the initial questionnaire was reviewed by 10 Saudi mothers, including 5 health science experts (two PhDs in clinical Nutrition, one PhD in Epidemiology, and two PhDs in Allied Health Sciences) alongside representative lay mothers. Subsequently, a pilot study was conducted with 30 participants who met the inclusion criteria (and were excluded from the main analysis) to evaluate survey feasibility and conciseness, and minor revisions were made based on their feedback.

The survey was administered via Google Forms, distributed through WhatsApp links. WhatsApp served exclusively as a broadcasting channel for the survey link. To prevent duplicate submissions, Google Forms Duplicate entries were prevented by restricting submissions to one per unique email address. Before accessing the survey items, participants were presented with an introductory page detailing the study’s purpose and the voluntary nature of participation. Participants were provided with brief standardized information about human milk banking and its clinical applications, followed by formal informed consent requirements before proceeding with questionnaire completion. The study was approved by the Ethics Committee of the College of Applied Medical Sciences, Taibah University (Approval no. 2023/169/112 MLT).

### Validity and reliability analysis

Item selection was guided by the Health Belief Model to capture maternal perceptions, religious considerations, and operational barriers regarding donor milk. Content and face validity were established using a 10-member expert and lay panel to ensure clarity, relevance, and cultural appropriateness of the items. Exploratory factor analysis (EFA) and internal consistency reliability testing using Cronbach’s alpha were not performed because the questionnaire was not designed as a unidimensional psychometric scale. Instead, it comprised discrete categorical items addressing distinct domains, including knowledge, perceptions, religious considerations, and situational willingness to donate or receive donor milk. As these items do not represent interchangeable indicators of a single underlying latent construct, factor analysis and internal consistency estimates were not methodologically appropriate for this instrument.

### Data analysis

The demographic characteristics of the participants and their perspectives on human milk banking and milk donation were presented as both frequencies and percentages. Statistical analysis was performed using SPSS version 20 (SPSS, Inc., Chicago, IL, USA). All data were subjected to descriptive statistics and normality tests prior to further analysis. Comparison of variables between women who supported HMB and those who did not was performed using the Chi-squared test or Fisher’s exact test, where expected cell counts were less than 5 for categorical variables. A significance level of *p* < 0.05 was used to determine statistical significance in all tests. *Post hoc* analysis was conducted using adjusted standardized residuals to identify cells contributing to significant associations. To control for multiple comparisons, Bonferroni correction was applied, and *adjusted p*-values were used to determine statistical significance. Logistic regression analysis was conducted to investigate the relationship between support for establishing a human milk bank (Dependent Variable) and demographics and other variables (Predictor Variables). Results are presented as Odds Ratios (OR) with 95% confidence intervals, where OR >1 indicates increased likelihood of support and OR <1 indicates decreased likelihood.

## Results

### Descriptive characteristics of study population

#### Sociodemographic characteristics

A total of 835 mothers were enrolled in the study (93.8% of 890 approached). Participants were predominantly aged 31–40 years (55.4%, *n* = 463), with smaller proportions aged 21–30 (17.6%, *n* = 147) and >40 years (26.9%, *n* = 225). Educational attainment included undergraduate degree holders (58.0%, *n* = 484), postgraduate education (30.9%, *n* = 258), and high school or lower (11.1%, *n* = 93). Employment status showed 57.5% (*n* = 480) were employed, 38.6% (*n* = 322) were housewives, and 4.0% (*n* = 33) were students. Geographic distribution was concentrated in the Western region (62.5%, *n* = 522), with a smaller proportion from Central (18.6%, *n* = 155), Northern (8.3%, *n* = 69), Eastern (6.8%, *n* = 57), and Southern (3.8%, *n* = 32) regions (Table 1). Family size ranged from one child (18.7%, *n* = 156) to more than four children (23.5%, *n* = 196), with 22.9% (*n* = 191) having two children and 20.8% (*n* = 174) having three children.

**Table 1:** Socio-demographic characteristics of the study sample (*n* = 835).

Variables	*n*	%
Age		
21–30	147	17.60
31–40	463	55.45
> 40	225	26.95
Educational level		
Below high school	19	2.28
High school	74	8.86
Undergraduate	484	57.96
Postgraduate	258	30.90
Occupation		
Employed	480	57.49
Housewife	322	38.56
Student	33	3.95
Residential region		
Western Region	522	62.52
Eastern Region	57	6.83
Northern Region	69	8.26
Southern Region	32	3.83
Central Region	155	18.56
No. of children		
One	156	18.68
Two	191	22.87
Three	174	20.84
Four	118	14.14
More than four	196	23.47

### Maternal feeding practices and breast Milk knowledge

Mixed feeding by maternal choice was the most common practice (59.6%, *n* = 298), followed by exclusive breastfeeding (24.0%, *n* = 200), and mixed feeding due to insufficient milk supply (14.5%, *n* = 121). A small proportion reported feeding with milk from relatives (0.5%, *n* = 4) or their own refrigerated pumped milk (1.4%, *n* = 12). Table 2 demonstrates that mothers practicing exclusive breastfeeding, duration varied substantially: 6–12 months was reported by 27.7% (*n* = 231), 12–18 months by 21.3% (*n* = 178), >18 months by 18.3% (*n* = 153), <3 months by 18.1% (*n* = 151), and <6 months by 14.6% (*n* = 122).

**Table 2:** Breastfeeding practices, knowledge, attitudes, and acceptance of human milk banking among Saudi mothers (*n* = 835).

Variables	*n*	%
The approach for feeding your child		
My own milk (natural breastfeeding)	200	23.95
My own milk and formula milk (my milk is not sufficient)	121	14.49
My own milk and formula milk (personal choice)	498	59.64
My own milk and milk from relatives	4	0.48
My own milk and my refrigerated pumped milk	12	1.44
The duration for exclusively breastfeeding		
< 3 months	151	18.09
< 6 months	122	14.61
6–12 months	231	27.66
12–18 months	178	21.32
> 18 months	153	18.32
Do you believe that breast milk is nutritionally superior to formula?		
Yes	786	94.13
No	20	2.40
I do not know	29	3.47
Do you believe that breast milk strengthens the baby’s immune system and therefore reduces the rate of infection?		
Yes	795	95.22
No	23	2.75
I do not know	17	2.03
What is your opinion on the legality of the concept of a Human Milk Bank?		
Permissible without any issues	15	1.80
Permissible if the donor is known	180	21.56
Permissible if no more than 4 feedings were taken from same mother	90	10.78
Impermissible due to milk kinship	261	31.26
Impermissible at all	87	10.42
I do not know	202	24.18
If your baby is born prematurely and in need of human milk, and you do not have enough milk, would you allow them to be provided with human milk from a milk bank?		
Yes	217	25.99
No	618	74.01
If you have an excess of milk that your baby does not need, is it possible for you to donate it to a milk bank?		
Yes	250	29.90
No	585	70.10
Do you believe that breast milk is beneficial only for the mother’s own child?		
Yes	243	29.10
No	592	70.90
Would you prefer to provide your child with formula even if there is a possibility of giving them donated milk?		
Yes	661	79.16
No	174	20.84
Is it possible for your stance on donated milk and milk banks to change if you learn more about the concept of a Human Milk Bank and the benefits of human milk for premature infants?		
Yes	165	19.76
No	351	42.04
Maybe	319	38.20
Would you consider donating your milk to a milk bank or giving your premature child milk from a milk bank if breastfeeding is documented on a government platform?		
Yes	330	39.52
No	505	60.48

Knowledge of the benefits of breast milk was generally high, as most mothers knew the nutritional superiority of breast milk (94.1%, *n* = 786) and its immune-protective properties (95.2%, *n* = 795). However, significant attitudinal gaps emerged when 79.2% (*n* = 661) of mothers preferred formula over donor human milk, and 29.1% (*n* = 243) believed breast milk benefits only their own child (Table 2).

### Association between HMB support and sociodemographic characteristics

Overall, 37.7% (*n* = 315) of mothers expressed support for HMB, while 62.3% (*n* = 520) did not support HMB. Supporting HMB was significantly associated with maternal age (*p* = 0.002) and number of children (*p* < 0.001). Mothers aged 21–30 years demonstrated the highest support rate (45.6%, *n* = 67), which declined progressively with advancing age: 31–40 years (39.8%, *n* = 184) and >40 years (28.5%, *n* = 64). Similarly, support decreased with increasing family size: mothers with one child showed the highest support (47.4%, *n* = 74), while those with >4 children showed the lowest (27.0%, *n* = 53). Support was independent of educational attainment (*p* = 0.880), geographic region (*p* = 0.451), and employment status (*p* = 0.065) (Table 3).

**Table 3:** Study sample characteristics stratified by support for human milk banking (*n* = 835).

Variables	Support of human milk banks (*n* = 315)	Non-support of human milk banks (*n* = 520)	Adjusted residual (Support)	*p*-value
Age				
21–30	67 (45.57)	80 (54.43)	2.2	0.002*
31–40	184 (39.75)	279 (60.25)	1.3	
> 40	64 (28.45)	161 (71.55)	−3.4**	
Educational level				
Less than high school	8 (42.10)	11 (57.90)	0.4	0.880
High school	25 (33.78)	49 (66.22)	−0.7	
Undergraduate	184 (38.02)	300 (61.98)	0.2	
Postgraduate	98 (37.98)	160 (62.02)	0.1	
Occupation				
Employed	168 (35.00)	312 (65.00)	−1.9	0.065
Housewife	137 (42.50)	185 (57.50)	2.3	
Student	10 (30.30)	23 (69.70)	−0.9	
Residential region				
Western Region	205 (39.27)	317 (60.73)	1.2	0.451
Eastern Region	25 (43.90)	32 (56.10)	1.0	
Northern Region	23 (33.33)	46 (66.66)	−0.8	
Southern Region	11 (34.40)	21 (65.60)	−0.4	
Central Region	51 (32.90)	104 (67.10)	−1.4	
No. of children				
One	74 (47.43)	82 (52.57)	2.8	<0.001*
Two	84 (43.98)	107 (56.02)	2.0	
Three	68 (39.08)	106 (60.92)	0.4	
Four	36 (30.50)	82 (69.50)	−1.7	
More than four	53 (27.04)	143 (72.96)	−3.5***	

*p < 0.05 for the overall Chi-square test.

**p < 0.008 and ***p < 0.005 (Bonferroni adjusted *post hoc* comparisons).

### Association between HMB support and feeding practices, knowledge, and attitudes toward breast Milk benefits

Support for HMB was not significantly associated with current feeding approach (*p* = 0.109) or duration of exclusive breastfeeding (*p* = 0.963). Among mothers practicing mixed feeding by choice, 36.9% supported HMB, compared to 37.5% of exclusively breastfeeding mothers and 37.2% of mothers with mixed feeding due to insufficient milk (Table 4).

**Table 4:** Breastfeeding knowledge and attitudes toward human milk benefits: associations with support for human milk banks (*n* = 835).

Variables	Support of human milk banks (*n* = 315)	Non-support of human milk banks (*n* = 520)	Adjusted Residual (Support)	*p*-value
The approach for feeding your child				
My own milk (natural breastfeeding)	75 (37.50)	125 (62.50)	−0.1	0.109
My own milk and formula milk (my milk is not sufficient)	45 (37.20)	76 (62.80)	−0.1	
My own milk and formula milk (personal choice)	184 (36.90)	314 (63.10)	−0.6	
My own milk and milk from relatives	2 (50.00)	2 (50.00)	0.5	
My own milk and my refrigerated pumped milk	9 (75.00)	3 (25.00)	2.7	
The duration for exclusively breastfeeding				
<3 months	54 (35.80)	97 (64.20)	−0.5	0.963
<6 months	49 (40.20)	73 (59.80)	0.6	
6–12 months	86 (37.20)	145 (62.80)	−0.2	
12–18 months	68 (38.20)	110 (61.80)	0.1	
> 18 months	58 (37.90)	95 (62.10)	0.1	
Do you believe that breast milk is nutritionally superior to formula?				
Yes	298 (37.90)	488 (62.1)	0.5	0.903
No	7 (35.00)	13 (65.0)	−0.3	
I do not know	10 (34.50)	19 (65.5)	−0.4	
Do you believe that breast milk strengthens the baby’s immune system and therefore reduces the rate of infection?				
Yes	306 (38.50)	489 (61.50)	2.0	0.130
No	5 (21.70)	18 (78.30)	−1.6	
I do not know	4 (23.50)	13 (76.50)	−1.2	

Knowledge of nutritional superiority (*p* = 0.903) and immune benefits (*p* = 0.130) of breast milk was not significantly associated with HMB support. Among mothers aware of nutritional benefits, 37.9% supported HMB compared to 35.0% of those unaware. Similarly, 38.5% of mothers acknowledging immune benefits supported HMB versus 21.7% of those who disagreed (Table 4).

### Association between HMB support and religious/legal opinions, clinical attitudes

A strong association existed between HMB support and mothers’ opinions regarding the legality of human milk banking (*p* < 0.001). Support rates varied dramatically across legality categories. Among participants who viewed HMB as permissible without restrictions, 86.7% (13/15) supported HMB. Among those who considered it permissible with no more than four feedings from the same mother, 83.3% (75/90) supported HMB. Participants who were uncertain about its legality showed 55.0% support for HMB (111/202). Among those who believed it should involve known donors, 50.0% (90/180) supported HMB. In contrast, participants with milk kinship concerns showed only 8.8% support (23/261), while those who viewed it as impermissible regardless of circumstances showed the lowest support at 3.4% (3/87) (Table 5).

**Table 5:** Associations of participants’ attitudes and perceptions of human milk banks with support for human milk banks (*n* = 835).

Variables	Support of human milk banks (*n* = 315)	Non-support of human milk banks (*n* = 520)	Adjusted residual (support)	*p*-value
What is your opinion on the legality of the concept of a Human Milk Bank?				
Permissible without any issues	13 (86.70)	2 (13.30)	3.9**	<0.001*
Permissible if the donor is known	90 (50.00)	90 (50.00)	3.8**	
Permissible if no more than 4 feedings were taken from same mother	75 (83.30)	15 (16.70)	9.5**	
Impermissible due to milk kinship	23 (8.80)	238 (91.20)	−11.6**	
Impermissible at all	3 (3.40)	84 (96.60)	−7.0**	
I do not know	111 (55.00)	91 (45.00)	5.8**	
If your baby is born prematurely and in need of human milk, and you do not have enough milk, would you allow them to be provided with human milk from a milk bank?				
Yes	200 (92.20)	17 (7.80)	–	<0.001*
No	115 (18.60)	503 (81.40)	–	
If you have an excess of milk that your baby does not need, is it possible for you to donate it to a milk bank?				
Yes	213 (85.20)	37 (14.80)	–	<0.001*
No	102 (17.40)	483 (82.60)	–	
Do you believe that breast milk is beneficial only for the mother’s own child?				
Yes	61 (25.10)	182 (74.90)	–	<0.001*
No	254 (42.90)	338 (57.10)	–	
Would you prefer to provide your child with formula even if there is a possibility of giving them donated milk?				
Yes	186 (28.10)	475 (71.90)	–	<0.001*
No	129 (74.10)	45 (25.90)	–	
Is it possible for your stance on donated milk and milk banks to change if you learn more about the concept of a Human Milk Bank and the benefits of human milk for preterm infants?				
Yes	138 (83.60)	27 (16.40)	13.6**	<0.001*
No	34 (9.70)	317 (90.30)	−14.2**	
Maybe	143 (44.80)	176 (55.20)	3.3**	
Would you consider donating your milk to a milk bank or giving your preterm child milk from a milk bank if breastfeeding is documented on a government platform?				
Yes	236 (71.50)	94 (28.50)	–	<0.001*
No	79 (15.60)	426 (84.40)	–	

*p < 0.05 for the overall Chi-square test.

**p < 0.001(Bonferroni adjusted post hoc comparisons).

Strong associations also existed between HMB support and acceptance of donor milk for preterm infants, willingness to donate, openness to education, and willingness to participate via a government platform (all *p* < 0.001). Among participants who indicated that they would accept donor milk for a preterm infant, 92.2% (200/217) supported HMB, compared with 7.8% (17/217) who did not. Similarly, among participants who indicated that they would donate excess breast milk, 85.2% (213/250) supported HMB, compared with 14.8% (37/250) who did not (Table 5). With respect to educational receptivity, 83.6% (138/165) of participants who indicated that their stance could change after learning more about HMB supported HMB, compared with 16.4% (27/165) who did not. If breastfeeding were documented on a government digital platform, 71.5% (236/330) of participants who indicated willingness to donate or receive donor milk supported HMB, compared with 28.5% (94/330) who did not.

### Sociodemographic and behavioral predictors

As shown in Table 6, mothers with one child were significantly more likely to support HMB compared to those with more than four children (OR = 2.060, 95% CI [1.049–4.044], *p* = 0.036). There were no significant differences observed for mothers with two, three, or four children compared to the reference group. Regarding employment status, students were significantly less likely to support HMB compared to housewives (OR = 0.299, 95% CI [0.108–0.827], *p* = 0.020). Employed mothers showed a trend toward lower support compared to housewives, though this did not reach statistical significance (OR = 0.674, 95% CI [0.446–1.017], *p* = 0.060). Table 6 demonstrates that maternal age, education, region, and feeding Practices: Age, educational level, residential region, feeding approach, and duration of exclusive breastfeeding were not independently predictive of HMB support (all *p* > 0.05).

**Table 6:** Multivariable analysis of factors associated with acceptance of establishing human milk bank in Saudi Arabia.

Variables	Comparison group category	OR	95% Confidence Interval	*p*-value
Age	Reference (> 40)			
	21–30	1.221	0.630 to 2.367	0.554
	31–40	1.166	0.710 to 1.916	0.544
Educational Level	Reference (Postgraduate)			
	Less than High School	1.584	0.457 to 5.487	0.468
	High School	1.737	0.778 to 3.878	0.178
	Undergraduate	1.386	0.906 to 2.122	0.133
Occupation	Reference (Housewife)			
	Student	0.299	0.108 to 0.827	0.020*
	Employed	0.674	0.446 to 1.017	0.060
No. of Children	Reference (more than four)			
	One	2.060	1.049 to 4.044	0.036*
	Two	1.618	0.867 to 3.020	0.130
	Three	1.476	0.802 to 2.715	0.211
	Four	1.137	0.600 to 2.156	0.694
Region of Residence	Reference (Central Region)			
	Western Region	1.442	0.888 to 2.340	0.139
	Eastern Region	1.482	0.677 to 3.241	0.325
	Northen Region	1.880	0.836 to 4.230	0.127
	Southern Region	2.021	0.690 to 5.917	0.199
The approach for feeding your child	Reference (My own milk and my refrigerated pumped milk)			
	My own milk	0.217	0.032 to 1.454	0.115
	My own milk and formula milk (my milk is not sufficient)	0.212	0.031 to 1.429	0.111
	My own milk and formula milk (personal choice)	0.225	0.035 to 1.436	0.115
	My own milk and milk from relatives	0.589	0.030 to 11.653	0.728
The duration for exclusively breastfeeding	Reference (> 18 months)			
	< 3 months	0.837	0.416 to 1.683	0.618
	3–6 months	0.872	0.440 to 1.726	0.693
	6–12 months	0.896	0.501 to 1.601	0.710
	12–18 months	0.791	0.446 to 1.403	0.422
Opinion on the legality of the concept of a human milk bank	Reference permissible without any issues			
	I do not know	0.186	0.039 to 0.876	0.033*
	Impermissible at all	0.005	0.001 to 0.035	<0.001*
	Impermissible due to milk kinship	0.014	0.003 to 0.068	<0.001*
	Permissible if no more than 4 feedings were taken from same mother	0.803	0.158 to 4.092	0.792
	Permissible if the donor is known	0.149	0.031 to 0.702	0.016*

*p < 0.05.

### Religious and legal predictors

Compared to mothers viewing HMB as “permissible without any issues,” all other legality opinions reduced support odds, with effect sizes shown in Table 6. Drastic reductions occurred among those viewing HMB as impermissible (99.5% lower odds) or citing milk kinship concerns (98.6% lower odds). Moderate reductions appeared among those requiring a known donor (85.1% lower odds) or expressing uncertainty (81.4% lower odds). Importantly, mothers accepting HMB if limited to 4 feedings per donor were not significantly less likely to support it (*p* = 0.792).

## Discussion

This study examined Saudi mothers’ knowledge, attitudes, and Islamic perspectives regarding the establishment of HMB in maternity hospitals. Overall, 37.7% of mothers supported HMB while 62.3% did not. Attitudes appeared to be shaped predominantly by religious-ethical considerations—specifically concerns regarding Islamic milk kinship rather than by maternal education level, geographic region, or general awareness of breastfeeding benefits. At the same time, a substantial minority expressed conditional support when identity documentation and kinship safeguards were assured, indicating a potential pathway for context-appropriate and religiously acceptable milk bank models in Saudi Arabia.

This pattern is notable considering the strong clinical rationale for DHM as the preferred alternative when a mother’s own milk is unavailable for preterm and very low birthweight infants. Evidence consistently shows that while formula feeding may accelerate short-term growth in preterm infants, it is associated with a higher risk of NEC and other serious morbidities compared to HM, which supports healthier gut microbiota and better overall outcomes (11, 14, 21, 30–32). International guidelines emphasize that safe human milk banking must undergo rigorous donor screening, standardized pasteurization procedures, robust quality control procedures and equitable access to DHM (5, 33, 34). Importantly, these safety and quality requirements are compatible with the religious and cultural contexts of Muslim-majority societies. Thus, several scholars have highlighted that comprehensive identity management and traceability mechanisms within milk banking systems can be structured to address concerns related to Islamic milk kinship, while maintaining international safety standards and the nutritional and clinical benefits of DHM (32, 35).

Our findings contribute to the growing evidence that local ethical and religious beliefs significantly influence the acceptance of DHM and milk banking. In our study, higher support was observed among younger women and those with fewer children, while education and region did not significantly predict attitudes (Table 3). This pattern suggests that younger, primiparous mothers may be more open to novel healthcare innovations and represent a key target demographic for initial educational initiatives. Interestingly, this inverse association with parity contrasts with findings from Turkey, where mothers with three or more children exhibited more positive attitudes toward milk banking than those with fewer children (27). This divergence highlights how local socio-cultural norms uniquely modulate demographic influences on health decision-making.

Notably, neither maternal education level nor geographic region was significantly associated with HMB support. This finding is particularly notable because global literature frequently identifies higher maternal education and urban residence as strong positive predictors of milk bank acceptance, driven by higher health literacy and clinical exposure (20, 36). In contrast, our results demonstrate that in Saudi Arabia, maternal hesitation is not driven by educational or socio-economic gradients, but by religious-ethical values regarding milk kinship that cut across all demographic strata equally.

In Muslim-majority countries, milk kinship emerges as the primary barrier to donor human milk (DHM) acceptance, rather than lack of knowledge about breastfeeding benefits (21, 30, 32). This was evident in our study, as support for milk banking was unrelated to feeding practices, exclusive breastfeeding duration, or awareness of breast milk’s nutritional and immunological advantages, which were already high in our study (Table 4). Even among mothers who acknowledged that breast milk is nutritionally superior and reduces infant infection rates, overall support for HMB did not substantially increase. These findings confirm that opposition to HMB stems from specific jurisprudential concerns rather than an informational deficit regarding infant nutrition.

Instead, decision-making was dominated by religious-ethical principles derived from Islamic jurisprudence (Fiqh), which establishes that milk-feeding creates marital prohibitions equivalent to biological kinship (23, 31). However, respondents demonstrated openness to reconsidering their stance when structural safeguards were introduced. Acceptability increased dramatically when presented with specific operational scenarios; a vast majority supported HMB when donor milk was limited to four or fewer feedings per donor, directly satisfying jurisprudential thresholds for kinship establishment and endorsed official, government-regulated platforms to track donor-recipient relationships (37). Similarly, when presented with the scenario of a critically ill preterm infant in need of milk, willingness to accept DHM rose substantially, even among initially hesitant mothers.

These conditional acceptance patterns validate culturally adapted milk banking models successfully implemented in other Muslim majority contexts. For instance, Malaysia’s national framework successfully integrated digital kinship tracking and Islamic jurisprudence into milk bank operations, achieving high public and scholar acceptance (38). Likewise, in Iran, mothers generally support donor milk usage when framed within clear religious rulings, despite limited initial formal knowledge (26). In contrast, maternal concerns in South Africa and Ethiopia focus predominantly on infection transmission and hygiene, reflecting differences in local health communication priorities (36, 39). Thus, while Western-style anonymous donation models face religious resistance in Islamic contexts, systems incorporating identity registration and joint scholar-clinician oversight overcome key ethical barriers (18, 20, 21, 27–29, 32, 36–42).

Translating these conditional acceptance factors into a functional HMB framework in Saudi Arabia relies on two interdependent pillars. Operationally, successful HMB demands integrated digital identity systems with secure kinship documentation registries to ensure compliance with Islamic requirements (21, 22, 30, 32). Governance-wise, formalizing collaborative structures involving Islamic scholars, pediatricians, neonatologists, and health authorities is essential to establish legitimacy and signal that milk banking is both clinically necessary and Islamically permissible (32, 40–42).

The Saudi Ministry of Health should prioritize co-development of integrated HMB frameworks with Islamic scholars, clinical experts, and community representatives. This includes establishing protocols for verifying milk kinship, integrating HMB into national neonatal guidelines aligned with international standards (4, 11, 14) and implementing centralized infrastructure with quality control oversight and donor screening protocols (5, 33, 34). Policy should limit DHM feedings to ≤4 per donor to comply with Fiqh-based thresholds (21, 24, 30, 32), and framing milk donation as altruistic Islamic charity will enhance sustainability (40, 42). Clinically, DHM should be prioritized for preterm and very low birthweight infants, where evidence of reduced necrotizing enterocolitis (NEC) risk and improved feeding tolerance is strongest (4, 11, 14). Donor human milk represents the evidence-based alternative to informal milk sharing, which lacks serological screening and carries documented risks of transmitting tuberculosis, hepatitis B and C, and HIV (2, 3, 14). Clear communication regarding these safety protocols is vital for public trust (18, 33, 34).

This study has several limitations. The retrospective cross-sectional design cannot establish causality between demographic factors and attitudes toward HMB. The online questionnaire may exclude mothers with limited internet access, possibly biasing the sample toward more educated and digitally connected respondents. Moreover, breastfeeding information was collected retrospectively based on mothers’ recall of past experiences, which may be subject to recall bias. The study is also subject to social desirability bias, particularly regarding religious and charitable obligations, which likely inflated support estimates and underreported barriers among religiously observant respondents. WhatsApp-based snowball sampling introduced selection and referral biases, as the sample was skewed toward university-educated participants and those residing in the Western region, exceeding the proportion of these demographics in the broader Saudi maternal population. Finally, the study did not collect data on clinical factors such as preterm birth, neonatal intensive care unit (NICU) admission, or neonatal complications, variables that likely significantly influence maternal attitudes toward HMB.

These limitations suggest several directions for future research. Stratified sampling strategies to achieve a more representative demographic distribution across educational levels and geographic regions. Importantly, future studies must collect data on clinical factors such as preterm birth, NICU admission, and neonatal complications, as these variables might significantly influence maternal attitudes toward HMB and donation intentions. Longitudinal studies would help establish temporal relationships between awareness, attitudes, and actual milk donation behavior. Additionally, qualitative research exploring the perspectives of religiously observant mothers and healthcare providers would illuminate the religious and clinical concerns that shape HMB implementation in Saudi Arabia.

## Conclusion

This study reveals that 38% of Saudi mothers support human milk banking, while 62% express concerns predominantly about milk kinship and safety/hygiene. These findings demonstrate that HMB implementation requires governance frameworks co-developed with religious scholars and clinicians to address milk kinship through secure donor-recipient registries, public education to address safety concerns, and integration into national neonatal guidelines for preterm and very low birthweight infants. Success depends on translating these insights into culturally responsive, clinically rigorous, and religiously informed practice in Saudi Arabia.

## Data Availability

The raw data supporting the conclusions of this article will be made available by the authors, without undue reservation.
