# Supplementary material for: Knowledge, perceptions, and support for human milk banking among Saudi mothers: a cross-sectional study
Source: Front Public Health. 2026 Sep 3;14:1914184. doi: 10.3389/fpubh.2026.1914184 (PMC13582417; doi:10.3389/fpubh.2026.1914184)
Supplement: Supplementary file 1 [file Table_1.docx]

| **No.** | **Variable** | **Question** | **Options / Scale** |
| --- | --- | --- | --- |
| 1 | Consent | Do you agree to participate in this survey? | - Yes - No |
| 2 | Age | What is your age? | - <20 - 21–30 - 31–40 - >40 |
| 3 | Education Level | What is your highest level of education? | - Illiterate - Literate - Primary - Middle - Secondary - University - Postgraduate |
| 4 | Occupation | What is your occupation? | - Housewife - Student - Employed |
| 5 | Number of Children | How many children do you have? | - 1 - 2 - 3 - 4 - More than 4 |
| 6 | Region | Which region of Saudi Arabia are you from? | - Eastern - Western - Central - Northern - Southern |
| 7 | Feeding Method | What feeding method do you use for your child? | - Breastfeeding only - Breastfeeding + Formula - Formula (insufficient milk) - Formula (personal choice) - Wet nurse (non-relative) - Wet nurse (relative) - Expressed milk (non-relative) - Expressed milk (relative) |
| 8 | Breastfeeding Duration | What is the usual duration of breastfeeding (exclusive)? | - <3 months - <6 months - 6–12 months - 12–18 months - >18 months - Not applicable |
| 9 | Perception (Nutrition) | Do you believe human milk is nutritionally better than formula? | - Yes - No - Don’t know |
| 10 | Perception (Immunity) | Do you believe human milk strengthens infant immunity and reduces infections? | - Yes - No - Don’t know |
| 11 | Awareness | Have you heard of a Human Milk Bank? | - Yes - No |
| 12 | Willingness to Donate | If you have excess milk, would you donate it to a milk bank? | - Yes - No |
| 13 | Barriers to Donation | If no, what are the reasons? | - Do not want milk kinship - Save milk for my child - Avoid medical tests - No time - Husband doesn’t agree - Prefer not to say |
| 14 | Acceptance (Receiving Milk) | If your premature baby needed milk, would you allow donor milk? | - Yes - No |
| 15 | Support for Milk Banks | Do you support establishing milk banks in hospitals? | - Yes - No |
| 16 | Religious Opinion | What is your opinion regarding milk banks from a religious perspective? | - Not permissible (milk kinship) - Permissible if donor identified - Permissible if mixed milk - Not permissible even if mixed - Completely unacceptable - Permissible without restriction - Don’t know |
| 17 | Knowledge (Storage) | Do you know how donor milk is stored in milk banks? | - Yes - No |
| 18 | Belief (Exclusive Benefit) | Do you believe breast milk benefits only the biological child? | - Yes - No |
| 19 | Preference | Would you prefer formula over donor milk even if donor milk is available? | - Yes - No |
| 20 | Reasons for Preference | If yes, why? | - Do not trust unknown donor - Affects child traits - Religious concerns - Husband refusal - Avoid milk kinship - Infection risk - Prefer not to say |
| 21 | Attitude Change | Would your opinion change if you learned more about milk banks? | - Yes - No - Maybe |
| 22 | Policy Acceptance | Would you donate or accept donor milk if breastfeeding relationships are officially documented (government system)? | - Yes - No |
